# Knowledge and Practice Regarding Abnormal Vaginal Discharge Among Adolescent Females in Riyadh City: An Observational Study

**DOI:** 10.7759/cureus.56719

**Published:** 2024-03-22

**Authors:** Helalah K Alenizy, May H AlQahtani, Sarah A Aleban, Reham I Almuwallad, Lolwah A Binsuwaidan, Durrah W Alabdullah, Asma E Althomali

**Affiliations:** 1 Obstetrics and Gynecology, Princess Nourah Bint Abdulrahman University, Riyadh, SAU; 2 Medicine, Princess Nourah Bint Abdulrahman University, Riyadh, SAU; 3 Medicine, King Saud University, Riyadh, SAU

**Keywords:** saudi arabia, practice, knowledge, prevalence, school-age girls, leucorrhea, vaginal discharge

## Abstract

Introduction

Vaginal discharge (VD) is a common condition that affects women during their childbearing years and often requires medical attention. It results from the physiological secretion of cervical and Bartholin's glands, as well as the shedding of vaginal epithelial cells caused by bacterial action in the vagina, which alters the acidic environment of the vagina. Experiencing vaginal symptoms is a common reason for seeking medical attention, especially among women during their reproductive years. This often leads to a visit to an obstetrician or a gynecologist. Accordingly, addressing such issues becomes even more crucial. The aim of this study is to assess the knowledge and practice regarding abnormal VD (AVD) among adolescent females in Riyadh City, Saudi Arabia.

Methods

The present study utilized a correlational cross-sectional survey methodology conducted in Riyadh City. The questionnaire was employed as the data collection instrument from November 2022 to November 2023. Eligibility for inclusion was limited to adolescent females and students living in Riyadh City, aged from 14 to 20 years. Electronic consent was obtained from participants aged 18 years and above, while consent from guardians was sought for those below 18 years. This sample size was determined with a minimum requirement of 500 participants, and 824 were involved. The questionnaire encompassed several sections, including demographic characteristics (gender, age, education, and menstruation history), history of AVD, knowledge regarding VD, and students' practices and behaviors related to VD. Cronbach’s alpha values for all the sections were more than 0.7. Data analysis was performed using statistical software, employing descriptive analysis, chi-square tests, and t-tests.

Results

A total of 824 girls were included, and their ages ranged from 14 to 20 years, with a mean age of (16 years ± 5) years old. Exactly 697 (84.6%) were high school students. Most of the study students (85.1%; 701) complained of an AVD at any point in their lives. Only 97 (11.8%) of the study students had a good knowledge level of VD. Higher age, marriage, late menarche, and seeking medical care for complaints of VD were the factors associated with a high knowledge level about VD (P<0.05). Additionally, 44.2% of school-age females sought medical care when experiencing AVD, with reasons including worsening symptoms over time and fear of serious diseases. However, a significant portion of participants opted for self-treatment using herbal remedies, medication from pharmacies, or leaving VD untreated, citing reasons such as perceiving it as a simple condition or fearing examination and disclosure.

Conclusion

In summary, the current study revealed that adolescent females demonstrate a sub-optimal level of knowledge regarding AVD. These findings are primarily observed among adolescent girls and individuals who exhibit a reluctance to seek appropriate medical intervention when having AVD.

## Introduction

Experiencing vaginal symptoms is a common reason for seeking medical attention, especially among women during their reproductive years. This often leads to a visit to an obstetrician or a gynecologist [1]. One such symptom is leucorrhoea, which refers to persistent and excessive vaginal discharge (VD) [2]. Leucorrhoea, a VD, is a common symptom of gynecological issues [3] and a significant burden of disease in developing countries [4]. Investigating VD is crucial as it affects a patient's personal, professional, and social life [5]. Most researchers use culture and microscopic studies to investigate the vaginal normal flora. These studies have shown that the dominance of several Lactobacillus species promotes vaginal health. These species maintain the vaginal pH at 4.5 by producing lactic acid, creating an acidic environment that is hostile to many bacteria [6].

Leucorrhoea is a discharge that can either be physiological or pathological. The physiological discharge comes from the secretions of cervical and Bartholin's glands [2,7]. On the other hand, pathological discharge is mainly caused by the replacement of the normal vaginal flora with bacteria [7]. The organisms that cause vaginitis are Gardnerella vaginalis, Trichomonas vaginalis, and Candida albicans [5]. A study conducted in Ghana showed that 66% of university students had vaginitis [8]. Meanwhile, a recent local study in Saudi Arabia revealed that the main symptom of patients with vaginitis and vaginal candida was a white, thick discharge [9]. Noninfectious discharge is caused by physical, mechanical, or chemical irritation [5]. Hormonal levels also influence leucorrhoea [4]. If left untreated, pathological discharge may lead to complications such as malignancy, pelvic inflammatory disease, and ectopic pregnancy. Therefore, early recognition and treatment are essential to decrease morbidity [7].

Data from several studies show differences in the prevalence of leucorrhoea. In India, the prevalence was recorded at 28.9%, while surveys conducted in Saudi Arabia showed a prevalence of 47.7% [1,10]. Two published studies in India reported a strong association between low socioeconomic status and leucorrhoea [11,12]. Similar to the Jatinangor study, it was found that the young age of marriage correlates with the incidence of leucorrhoea. This is due to premature sexual activity, which exposes infectious organisms to the undeveloped cervical epithelium [13]. Investigating the prevalence, knowledge, practice, and associated factors regarding abnormal VD in our specific population will help us understand the local burden of the condition and identify potential risk factors. By addressing these aspects, we aim to contribute to the existing knowledge, enhance preventive strategies, and improve the overall reproductive health and well-being of adolescent females.

## Materials and methods

A descriptive cross-sectional survey was conducted in Riyadh City using electronic questionnaires distributed through multiple data collectors from November 2022 to November 2023. Inclusion and exclusion criteria were employed to ensure the appropriate selection of participants. Adolescent females aged from 14 to 20 years and living in Riyadh City were eligible for inclusion in the study. On the other hand, those who refused to participate, non-Arabic speakers, and with incomplete survey answers were excluded. Consent to participate in the questionnaire was obtained from all participants aged 18 years and above. For participants younger than 18 years, consent from their guardians was sought prior to their involvement in the study. The sample size was determined using the G-power (The G*Power Team, Germany) analysis program, which recommended a minimum of 500 participants for a study with a 95% confidence level and a power of study of 95%. For this study, a total of 824 respondents were included. The survey is self-structured. The validity of the questionnaire was assessed, and Cronbach's alpha values for all sections were found to be satisfactory, being greater than (0.7).

Convenient sampling was employed to select participants from different educational institutes, and data collection was carried out through multiple data collectors till no more answers were added. The questionnaire was designed in Arabic and contains three sections. The first section was about socio-demographic and menstruation data. The second section covered exploring and assessing the knowledge regarding VD. The third section focused on exploring the practice and various behaviors towards abnormal vaginal discharge (AVD). Data were collected, coded, and directly analyzed.

Data analysis

The data were collected, reviewed, and then fed to the Statistical Product and Service Solutions (SPSS, version 24; IBM SPSS Statistics for Windows, Armonk, NY). All statistical methods used were two-tailed with an alpha level of 0.05 considered significant if the P value is less than or equal to 0.05. The overall knowledge level regarding AVD was assessed by summing up discrete scores for different correct knowledge items. The overall knowledge score was categorized as a poor level if the student's score was less than 60% of the overall score, and a good level of knowledge was considered if the participant's score was 60% or more of the overall score. Descriptive analysis was done by describing frequency distribution and percentage for study variables including students' socio-demographic and medical data including menstrual history. Additionally, the frequency distribution of the students' knowledge about AVD, their practice, and preventive behaviors were tabulated, and their overall knowledge level was graphed. Cross-tabulation for showing factors associated with students' overall knowledge level about AVD using the Pearson chi-square test for significance and Fisher's exact test if there were small frequency distributions.

## Results

The study comprised 824 female participants, with ages spanning from 14 to 20 years and a mean age of 16 ± 5 years. Among them, 697 (84.6%) were enrolled in high school, while 127 (15.4%) were attending intermediate school. Furthermore, the majority of the participants (735, 89.2%) were of Saudi nationality. In terms of financial status, exactly 507 (61.5%) possessed a monthly income that met their basic needs, while 200 (24.3%) had an income exceeding their requirements. Conversely, 117 (14.2%) of participants faced financial insufficiency. The overwhelming majority of the study participants (779, 94.5%) were single. Regarding menstruation patterns, 809 (98.2%) experienced their first menstrual period, with 265 (32.8%) individuals initiating menstruation between the ages of nine and 11 years and 479 (59.4%) experiencing it between the ages of 12 and 14 years. With regard to living arrangements, 712 (86.4%) of the participants resided with their parents, and 755 (91.6%) reported that their parents were married (Table 1).

**Table 1:** Socio-demographic characteristics of school-age females in Saudi Arabia

Socio-demographic	No	%
Age in years		
14-15	152	18.4%
16-17	342	41.5%
18-19	186	22.6%
20	144	17.5%
Nationality		
Saudi	735	89.2%
Non-Saudi	89	10.8%
Grade		
Intermediate school	127	15.4%
High school	697	84.6%
Income		
Insufficient & loan	58	7.0%
Loan	59	7.2%
Just sufficient	507	61.5%
More than sufficient	200	24.3%
Marital status		
Single	779	94.5%
Married	45	5.5%
Have you had your first period?		
Yes	809	98.2%
No	15	1.8%
At which age you experienced the first period?		
9-11	265	32.8%
12-14	479	59.4%
15-17	63	7.8%
Living with		
Both parents	712	86.4%
Mother	67	8.1%
Father	23	2.8%
Relatives	22	2.7%
Parents' marital status?		
Married	755	91.6%
Divorced	69	8.4%

Table 2 provides insights into the history of VD among adolescent females in Riyadh City. The majority of the study's participants (701, 85.1%) reported experiencing AVD at some point in their lives. Among them, 303 (43.2%) described the discharge as moderate, while 294 (41.9%) reported minimal discharge. The characteristics of the discharge varied among the participants. About 323 (46.1%) described it as clear and transparent. Additionally, 333 (47.5%) of them prescribed it as cheesy and whitish in color, and 45 (6.4%) individuals described it as greenish-yellow. Approximately 329 (46.9%) of those with VD reported it to be malodorous. In terms of the duration of experiencing VD, 515 (73.5%) experienced VD for less than 15 days. Among them, 338 (48.2%) described the discharge as thin and mucoid, while 256 (36.5%) characterized it as clear. Approximately 276 (39.4%) of students with VD used sanitary pads, and 104 (37.7%) changed their pads twice daily. Regarding the relationship between VD and the menstrual period, 344 (49.1%) stated that the discharge was unrelated to their period. However, 212 (30.2%) experienced the discharge before their period, and 132 (18.8%) had it after their period. The most commonly reported associated symptoms were vulvar itching (171, 24.4%) and lower abdominal pain (125, 17.8%). Interestingly, 371 (52.9%) individuals did not experience any associated symptoms.

**Table 2:** Vaginal discharge history among school-age females in Riyadh City

Vaginal discharge	n (824)	%
Have you ever complained of any abnormal vaginal discharge at any point in your life?		
Yes	701	85.1%
No	123	14.9%
The amount of abnormal vaginal discharge daily? (n=701)		
Moderate	303	43.2%
Minimal	294	41.9%
Copious	58	8.3%
Heavy	46	6.6%
What is the color of the discharge? (n=701)		
Clear, transparent	323	46.1%
Cheesy whitish	333	47.5%
Greenish yellow	45	6.4%
Malodorous of the discharge? (n=701)		
Yes	329	46.9%
No	372	53.1%
What was the duration of the discharge existence? (n=701)		
< 15 days	515	73.5%
> 15 days	186	26.5%
What was the nature of the discharge? (n=701)		
Thin mucoid	338	48.2%
Clear	256	36.5%
Thick curdy	79	11.3%
Frothy	28	4.0%
Do you use sanitary pads once you have the abnormal vaginal discharge? (n=701)		
Yes	276	39.4%
No	425	60.6%
If yes, how many pads do you change per day? (n=276)		
Once daily	81	29.3%
Twice daily	104	37.7%
Three times and more	91	33.0%
Relation to period? (n=701)		
Before period	212	30.2%
After period	132	18.8%
During period	13	1.9%
Not related to the period	344	49.1%
Associated symptoms? (n=701)		
Vulvar itching	171	24.4%
Lower abdominal pain	125	17.8%
Dysuria	20	2.9%
Painful ulcers/rash	14	2.0%
None of these	371	52.9%

Table 3 presents the knowledge regarding abnormal VD among school-age females in Riyadh City. Regarding the criteria of AVD, 589 (71.5%) reported that a woman may experience VD at any point in her life, and 405 (49.2%) recognized that clear, non-offensive discharge that varies with the menstrual cycle is a normal physiological secretion. Furthermore, 364 (44.2%) individuals were aware that white or colored VD may be a sign of reproductive tract infections. Considering the causes of AVD, 563 (68.3%) acknowledged that women aged between 15 and 52 years have normal physiological vaginal secretions. Moreover, 473 (57.4%) understood that VD may vary in color, odor, and consistency depending on the cause, and 469 (56.9%) recognized that AVD may be caused by bacteria or fungus infections. In terms of risk factors associated with AVD, 367 (44.5%) knew that wearing tight underwear continuously may increase the incidence of abnormal discharge. On the other hand, 276 (33.5%) reported that personal hygiene of sensitive areas has nothing to do with the onset of AVD. Furthermore, 160 (19.4%) acknowledged that poorly controlled diabetes may increase the incidence of vaginal infections and the observation of strange VD. Regarding the treatment of AVD, exact of 522 (63.3%) were aware that the most appropriate treatment for AVD is by counseling an expert doctor. Additionally, 359 (43.6%) understood that the duration of treatment of vaginal infection causing abnormal discharge will depend on the cause itself. However, 242 (29.4%) believed that AVD can be treated by using vaginal douching.

**Table 3:** Knowledge regarding abnormal vaginal discharge among school-age females in Riyadh City AVD: Abnormal vaginal discharge

Knowledge items	Yes		No		Don't know	
	n (824)	%	n (824)	%	n (824)	%
Treatment of AVD						
The most appropriate treatment of abnormal vaginal discharge is by counseling an expert doctor.	522	63.3%	35	4.2%	267	32.4%
Abnormal discharge in color, consistency or odor treated perfectly with herbs and household mixtures.	88	10.7%	378	45.9%	358	43.4%
Abnormal vaginal discharge caused by vaginal infection has no cure?	51	6.2%	429	52.1%	344	41.7%
The duration of treatment of vaginal infection causing abnormal discharge will depend on the cause itself.	359	43.6%	61	7.4%	404	49.0%
Abnormal vaginal discharge can be treated by using vaginal douche.	242	29.4%	179	21.7%	403	48.9%
Risk factors of AVD						
Personal hygiene of sensitive areas has nothing to do with the onset of abnormal vaginal discharge.	276	33.5%	317	38.5%	231	28.0%
Poorly controlled diabetes may increase the incidence of vaginal infections and the observation of strange vaginal discharge?	160	19.4%	42	5.1%	622	75.5%
Wearing tight underwear continuously may increase the incidence of abnormal discharge.	367	44.5%	103	12.5%	354	43.0%
Criteria of AVD						
The presence of odorless white vaginal discharge may indicate a fungal infection.	192	23.3%	131	15.9%	501	60.8%
All the types of vaginal discharge are considered to be pathological conditions.	85	10.3%	437	53.0%	302	36.7%
A woman may experience vaginal discharge at any point in her life.	589	71.5%	39	4.7%	196	23.8%
A clear, non-offensive discharge that varies with the menstrual cycle is a normal physiological secretion.	405	49.2%	35	4.2%	384	46.6%
White or colored vaginal discharge may be a sign of reproductive tract infections.	364	44.2%	79	9.6%	381	46.2%
Causes of AVD						
The most common cause of abnormal vaginal discharge is genetic causes.	119	14.4%	184	22.3%	521	63.2%
Women aged between 15-52 years have a normal physiological vaginal secretion.	563	68.3%	33	4.0%	228	27.7%
Vaginal discharge may vary in color, odor and consistency depending on the cause.	473	57.4%	33	4.0%	318	38.6%
Repetitive use of vaginal douching may lead to the occurrence of abnormal vaginal discharge.	316	38.3%	80	9.7%	428	51.9%
There is no relation between the prolonged use of some medications like antibiotics or steroids and the occurrence of AVD.	119	14.4%	175	21.2%	530	64.3%
The abnormal vaginal discharge may cause by bacteria or fungus infections.	469	56.9%	46	5.6%	309	37.5%

**Figure 1:**
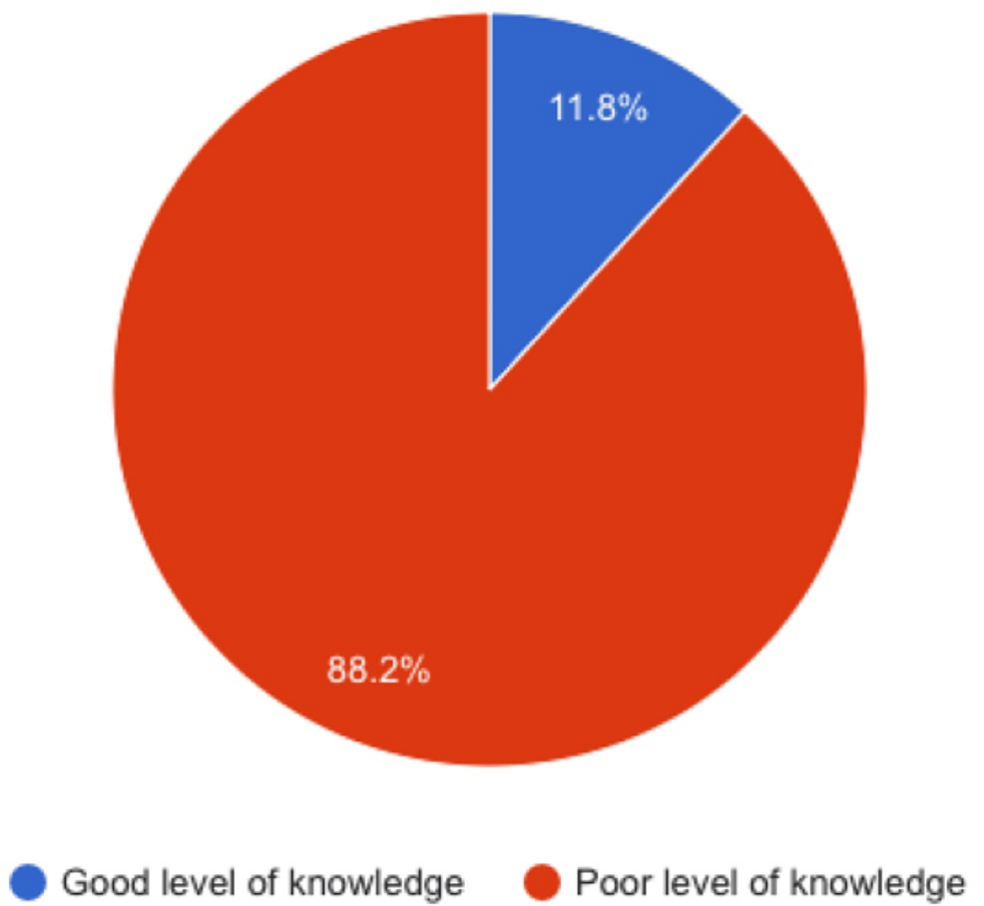
Overall knowledge regarding abnormal vaginal discharge among school-age females in Riyadh City The blue sector in the pie chart represents 727 (88.2%) of participants who exhibit a poor level of knowledge regarding abnormal vaginal discharge. The brown sector in the pie chart signifies 97 (11.8%) of participants with a good level of knowledge regarding abnormal vaginal discharge.

Figure 1 presents the overall knowledge regarding AVD among school-age females in Riyadh City. Only 97 (11.8%) of the study students had an overall good knowledge level about VD, while 727 (88.2%) had a poor knowledge level.

Table 4 presents the behaviors while having VD among adolescent females in Riyadh City. Exactly 364 (44.2%) seek medical care when they complain of any symptoms in the genital area. The most reported reasons were the case became worse with time (168, 46.2%) and fear of serious diseases (149, 40.9%). A total of 135 (16.4%) use herbal remedies and household mixtures to treat AVD when experiencing it, 116 (14.1%) treat AVD with medication obtained from a pharmacy instead of consulting a doctor, and 460 (55.8%) leave VD on until it subsides spontaneously. As for medication use, 170 (20.6%) use of over-the-counter medications such as vaginal douche, and 140 (82.4%) were improved. The most reported reasons for not seeking medical advice were thinking it is simple and normal and does not need medical attention (159, 34.6%), being afraid of revealing and examining the genitalia (106, 23%), having no one to explain to (63, 13.7%), and busy and ignored it (47, 10.2%).

**Table 4:** Behaviors while having vaginal discharge among school-age females in Riyadh City

Behaviors while having VD	n (824)	%
Do you seek medical care when you complain of any symptoms in the genitalia area?		
Yes	364	44.2%
No	460	55.8%
If yes, what was the reason for seeking medical care? (n=364)		
Fear of serious diseases	149	40.9%
The case becomes worse with time	168	46.2%
Inability to tolerate abnormal symptoms	23	6.3%
Previous good experience with medical treatment	24	6.6%
Do you use herbal remedies and household mixtures to treat abnormal vaginal discharge when you're experiencing it?		
Yes	135	16.4%
No	689	83.6%
Do you treat abnormal vaginal discharge with medication obtained from a pharmacy instead of consulting a doctor?		
Yes	116	14.1%
No	708	85.9%
When you have a complaint about abnormal vaginal discharge, do you leave it on until it subsides spontaneously?		
Yes	460	55.8%
No	364	44.2%
Do you use medications over the counter like vaginal douche?		
Yes	170	20.6%
No	654	79.4%
If yes, does it improve? (n=170)		
Yes	140	82.4%
No	30	17.6%
If you don’t seek medical examination, then the reason is (n=460)		
It is simple and normal and don’t need examination	159	34.6%
Afraid of revealing and examining the genitalia	106	23.0%
Have no one to explain to	63	13.7%
Busy and ignore it	47	10.2%
Lack of awareness or knowledge	45	9.8%
It is relived with home remedies	23	5.0%
Social stigma	17	3.7%

Table 5 presents the preventive practice to prevent abnormal discharge among school-age females in Riyadh City. A total of 320 (38.8%) students have prior knowledge of the best cleaning routine for sensitive areas. Exactly 406 (49.3%) students use water only routinely in hygienic practice to wash and clean the genitalia, 255 (30.9%) use water and soap, and 163 (19.8%) use medical vaginal douche. A total of 751 (91.1%) apply hand washing before and after cleaning the genital area. The preferred underwear was cotton (499, 60.6%) and cotton and polyester (278, 33.7%). Additionally, 648 (78.6%) use comfortable underwear, 359 (43.6%) change their underwear daily, and 232 (28.2%) change every two days. Exactly 403 (48.9%) remove genital hair every 20 days, but 322 (39.1%) do it every month.

**Table 5:** Preventive practice to prevent abnormal discharge among school-age females in Riyadh City

Preventive practice to prevent abnormal discharge	n (824)	%
Do you have any prior knowledge of the best cleaning routine for sensitive areas?		
Yes	320	38.8%
No	504	61.2%
Your routinely hygienic practice to wash and clean the genitalia is		
Using water only	406	49.3%
Using water and soap	255	30.9%
Using medical vaginal douche	163	19.8%
Do you apply hand washing before and after cleaning the genital area?		
Yes	751	91.1%
No	73	8.9%
The preferred underwear to wear is		
Cotton	499	60.6%
Polyester	47	5.7%
Cotton and polyester	278	33.7%
The preferred underwear to wear is		
Comfortable	648	78.6%
Tight	98	11.9%
Wide	78	9.5%
Changing underwear interval is		
More than one per day if needed	189	22.9%
Everyday	359	43.6%
Every 2 days	232	28.2%
Every week	44	5.3%
The time interval of genitalia hair removal		
Every 20 days	403	48.9%
Every month	322	39.1%
Every 2 months	56	6.8%
More than 2 months	43	5.2%

Table 6 presents the factors associated with the overall knowledge level about AVD among school-age females in Riyadh City. Exactly 20 (16.7%) of students aged 20 years or more had an overall good knowledge of VD compared to 11 (7.2%) of others aged 14-15 years with recorded statistical significance (P=0.015). Additionally, 14 (31.1%) of married students had an overall good knowledge versus 83 (10.7%) of single students (P=0.001). Good knowledge about VD was detected among 12 (19%) of students who experienced the first period at 15-17 years of age in comparison to 24 (9.1%) of those who had the period at the age of 9-11 years (P=0.048). Similarly, 61 (16.8%) of students who sought medical care when they complained of any symptoms in the genitalia area had good knowledge about VD versus 36 (7.8%) of others who did not (P=0.001).

**Table 6:** Factors associated with the overall knowledge level about abnormal vaginal discharge among school-age females in Riyadh City P: Pearson X2 test; ^: Exact probability test; * P < 0.05 (significant)

Factors	Overall knowledge level				p-value
	Poor		Good		
	n (824)	%	n (824)	%	
Age in years					.015*
14-15	141	92.8%	11	7.2%	
16-17	309	90.4%	33	9.6%	
18-19	157	84.4%	29	15.6%	
20+	120	83.3%	24	16.7%	
Nationality					.868
Saudi	648	88.2%	87	11.8%	
Non-Saudi	79	88.8%	10	11.2%	
Grade					.776
Intermediate school	113	89.0%	14	11.0%	
High school	614	88.1%	83	11.9%	
Income					.251^
Insufficient & loan	53	91.4%	5	8.6%	
Income	50	84.7%	9	15.3%	
Enough	454	89.5%	53	10.5%	
Enough and save	170	85.0%	30	15.0%	
Marital status					.001*
Single	696	89.3%	83	10.7%	
Married	31	68.9%	14	31.1%	
Had the first period?					.318^
Yes	715	88.4%	94	11.6%	
No	12	80.0%	3	20.0%	
Age of experiencing the first period					.048*
9-11	241	90.9%	24	9.1%	
12-14	421	87.9%	58	12.1%	
15-17	51	81.0%	12	19.0%	
History of complaining from any abnormal vaginal discharge at any point of your life					.452
Yes	616	87.9%	85	12.1%	
No	111	90.2%	12	9.8%	
History of seeking for medical care when you complain of any symptoms in the genitalia area					.001*
Yes	303	83.2%	61	16.8%	
No	424	92.2%	36	7.8%	

## Discussion

The promotion of reproductive health information is an essential component of preventing reproductive health issues among teenagers, particularly in developing countries [14]. Recently, a significant number of young people have suffered from leucorrhoea, which hinders their ability to grow and develop to their full potential [15]. Despite this, many people remain unaware and ignorant about how to prevent leucorrhea. Such ignorance poses a threat to their health not only in the short term but also in the long run [16]. Many school-age girls lack knowledge about leucorrhea, which can lead to health problems. It is important to provide information about the prevention, management, and maintenance of reproductive organs. However, it is not always possible for health workers to participate in every reproductive health program in schools and receive appropriate education on the matter.

The current study aimed to identify the level of knowledge about AVD among adolescent girls and assess practices regarding the prevention and management of VD. Regarding the frequency of experiencing VD, the current study revealed that more than three-fourths of the girls experienced AVD at any point in their lives mainly in minimal to moderate amounts. Most of the girls reported that VD was either clear or cheesy white, but less than half said it was malodorous. Additionally, about three-fourths experienced VD for less than two weeks, which was mainly unrelated to the period. More than half of them also said that they had no associated symptoms with VD, but some of them experienced vulvar itching and low abdominal pain. A much lower prevalence was reported by Guntoory et al. [10] in India with 28.9%. Its prevalence was found to be higher in the younger age group. It was also lower than reported in other studies by Singh et al. (29%) and (27.5%) [12,17]. In Egypt, Khadawardi [7] documented increased vaginal secretions during pregnancy reported in 73.9% of cases. As regards the characteristics of secretions, 41.9% had white translucent secretions, the majority of cases (72.1%) had odorless secretions, and 26.1% of cases had abnormal secretions due to bacterial infection and fungal infection (24.7%). In Nigeria, Uwakwe et al. [18] reported that 55.6% of women had AVD, and 73.3% among pregnant women. Most of the women had whitish VD (76.3%), and 49.6% had experienced foul and fish-smelling discharges. In Saudi Arabia, the incidence of AVD was 47.7%, which is much lower than reported in the current study of girls. The discharge was clear white in 44.3%, cheesy whitish in 35.3%, and yellow in 20.5% [19]. In Riyadh, Saudi Arabia, a study among 2,719 females over six months found that only 175 (6.4%) complained of VD [20]. Similar to the current study as most of the girls had a symptomatic VD, Li et al. [21] found that 21 women with AVD may not report it unless it significantly affects daily activities.

Regarding the current study of girls' knowledge about AVD, it was found that nearly one-tenth of the girls had good knowledge about the disorder. The most known criteria of VD were that a woman may experience VD at any point in her life. Clear, non-offensive discharge that varies with the menstrual cycle is a normal physiological secretion, and white or colored VD may be a sign of genital tract infections. Considering the causes of AVD, women aged between 15 and 52 years have normal physiological vaginal secretion; VD may vary in color, odor, and consistency depending on the cause; and AVD may be caused by bacteria or fungus infections, which were the most reported. The most known risk factors include wearing tight underwear continuously, which may increase the incidence of abnormal discharge; personal hygiene of the genital area has nothing to do with the onset of AVD; and poorly controlled diabetes may increase the incidence of vaginal infections and the observation of strange VD. About two-thirds of the study females know that the most appropriate treatment of AVD is by counseling an expert doctor, but less than half of them told that the duration of treatment for vaginal infection causing abnormal discharge will depend on the cause itself. Old age with late menstruation onset and seeking medical consultation were the most significant predictors for girls' knowledge level. Similar findings were reported by Rakhmilla et al. [22], as the results show that school-age girl’s knowledge about leucorrhoea is low. Similarly, Abdelmoneam et al. [23] revealed that women had unsatisfactory knowledge. On the other hand, Eram et al. [24] reported that all women know that the cause of VD is weakness and heat. Additionally, 87% of women said the cause was due to backache, and 93% of women reported the cause to be the melting of bones. The effects of VD, as reported by the majority of women, were weakness (100%), backache (100%), pain in the lower abdomen (37%), body ache (81%), and pallor (75%).

With regard to girls' practice, the current study revealed that less than half seek medical care when they complain of any symptoms in the genital area mainly due to fear of being worse with time and fear of serious diseases. Using herbal remedies and household mixtures to treat AVD was infrequent, but medication obtained from a pharmacy instead of consulting a doctor was reported. On the other hand, more than half of the girls with VD waited for it to subside spontaneously. Only one-fifth of the girls use over-the-counter medications, such as vaginal douching, and most of them were improved. The most reported reasons for not seeking medical advice were thinking it is simple and normal and does not need examination and being afraid of revealing and examining the genitalia. Considering preventive practices, more than one-third have prior knowledge of the best cleaning routine for sensitive areas. About half of the girls use water only and have routine hygienic practices to wash and clean the genitalia; about one-third use water and soap; and one-fifth use medical vaginal douche. The vast majority apply hand washing before and after cleaning the genital area. The preferred underwear was cotton or cotton and polyester. About half of the girls remove genital hair every 20 days, but 322 (39.1%) do every month. Bro [25] found that fear of having a serious disease or a sexually transmitted disease was the reason for the visit to the general practitioner in more than half of women in Denmark. Zaher et al. [26] documented that more than half of their study women did not consider VD as a serious problem that need a medical examination. A study [27] also found that most women expressed that they use home remedies before consulting a doctor for AVD. Educational campaigns and appropriate teaching materials are essential in improving adolescents' knowledge of AVD. As they transition into young adulthood, it is crucial for adolescents to understand the normal variations in VD and the potential warning signs of infections or other health issues. By integrating this information into the curriculum, educators can empower young people to take control of their reproductive health and seek medical attention when necessary. This not only promotes overall well-being and prevents the spread of sexually transmitted infections but also fosters a culture of open communication and destigmatizes discussions about reproductive health. Ultimately, providing adolescents with accurate information about VD can help them make informed decisions about their bodies and empower them to prioritize their health and well-being [28].

Limitations

First, although the study revealed unsatisfactory knowledge regarding abnormal vaginal discharge among school-age girls, the study was conducted only in Riyadh City, and this is considered a limitation as it is not representative of other regions in Saudi Arabia. Therefore, the results cannot be generalized to other regions in the country.

Second, our data were gathered via a survey that was restricted to female teenagers with the approval of their guardians and did not include non-Arabic-speaking people. The limitation was that we took time to obtain consent from the guardians before distributing the survey questionnaires.

Because data collection was done through the completion of online questionnaires, there may be communication barriers between researchers and respondents during the research process.

## Conclusions

In conclusion, the current study revealed that adolescent girls showed an unsatisfactory level of knowledge regarding AVD, mainly young-aged girls and those who did not seek medical care for any genital area symptoms previously. This was irrespective of the high-frequency rate of experiencing AVD but was mainly asymptomatic. On the other hand, girls reported practicing mainly preventive measures was satisfactory, but those using prescribed medications were below average. More effort should be paid to improve girls' knowledge, attitude, and safe practice toward AVD mainly through health education campaigns and study curricula.

## Appendices

Questionnaire (three sections)

**First section: sociodemographic data**

Age: 14-15 / 16-17/ 18-19 / 20 

Nationality: Saudi / Non-Saudi

Education: Middle school / High school

Family income: Enough / Enough and save / Not enough / Not enough and debt 

Marital status: Single / Married 

Have you had your first period? Yes / No

At which age you experienced your first period? 9-11 / 12-14 / 15-17

The housing status: Living with both parents / Living with other relatives / Living with the father only / Living with the mother only 

Parents' marital status: Married / Divorced

Vaginal discharge history

1. Have you ever complained of any abnormal vaginal discharge at any point in your life?

Yes

No

2. What was the duration of the discharge existence?

Less than 15 days

More than 15 days

3. What was the nature of the discharge?

Frothy

Thick curdy

Thin mucoid

Clear

4. Do you use sanitary pads once you have the abnormal vaginal discharge? 

Yes 

No

5. If yes, how many pads do you change per day?

Once daily 

Twice daily 

Three times and more

6. If no, how do you estimate the amount of the abnormal vaginal discharge daily?

Copious 

Minimal

Moderate

7. What is the color of the discharge?

Greenish-yellow

Cheesy whitish

Clear, transparent

8. Malodorous of the discharge?

Absent

Present

9. Relation to period?

After

Before

Continues over the cycle

10. Associated symptoms?

Lower abdominal pain

Vulvar itching

Dysuria

Painful ulcers over genitalia

Vaginal rash

**Knowledge regarding vaginal discharge: Yea, No, I don’t know**

- The most appropriate treatment of abnormal vaginal discharge is by counseling an expert doctor.

- Abnormal discharge in color, consistency or odor treated perfectly with herbs and household mixtures.

- Abnormal vaginal discharge caused by vaginal infection has no cure.

- The duration of treatment of vaginal infection causing abnormal discharge will depend on the cause itself.

- Abnormal vaginal discharge can be treated by using vaginal douche. 

- Personal hygiene of sensitive areas has nothing to do with the onset of abnormal vaginal discharge.

- Poorly controlled diabetes may increase the incidence of vaginal infections and the observation of strange vaginal discharge?

- Wearing tight underwear continuously may increase the incidence of abnormal discharge.

- The presence of odorless white vaginal discharge may indicate a fungal infection.

- All the types of vaginal discharge are considered to be pathological conditions.

- A woman may experience vaginal discharge at any point in her life.

- A clear, non-offensive discharge that varies with the menstrual cycle is a normal physiological secretion.

- White or colored vaginal discharge may be a sign of reproductive tract infections.

- The most common cause of abnormal vaginal discharge is genetic causes.

- Women aged between 15-52 years have a normal physiological vaginal secretion.

- Vaginal discharge may vary in color, odor and consistency depending on the cause.

- Repetitive use of vaginal douche may lead to the occurrence of abnormal vaginal discharge.

- There is no relation between the prolonged use of some medications like antibiotics or steroids and the occurrence of AVD.

- The abnormal vaginal discharge may be caused by bacteria or fungus infections.

**Behaviors while participants having discharge**

1. Do you seek medical care when you complain of any symptoms in the genitalia area (itching, abnormal discharge)?

Yes

No

2. Do you seek medical treatment for abnormal vaginal discharge from a specialist doctor when you complain about it? 

Yes

No

3. If yes, what was the reason for seeking medical care

Fear of serious diseases

Inability to tolerate abnormal symptoms ​​​​

The case becomes worse with time

Previous good experience with medical treatment

4. Do you use herbal remedies and household mixtures to treat abnormal vaginal discharge when you're experiencing it? 

Yes

No

5. Do you treat abnormal vaginal discharge with medication obtained from a pharmacy instead of consulting a doctor?

Yes 

No 

6. When you have a complaint about abnormal vaginal discharge, do you leave it on until it subsides spontaneously?

Yes 

No

7. Do you use over-the-counter medications like vaginal douche?

Yes

No

8. If yes, does it improve?

Yes

No

9. If you don’t seek a medical examination, then the reason is

Social stigma

It is simple and normal and don’t need examinations

Lack of awareness or knowledge

Afraid of revealing and examining the genitalia

Have no one to explain to

Busy and ignore it

It is relived with home remedies

**Preventive practices to prevent abnormal discharge**

10. Do you have any prior knowledge of the best cleaning routine for sensitive areas?

Yes

No 

11. Your routinely hygienic practice of washing and cleaning the genitalia is

Using water only 

Using water and soap 

Using medical vaginal douche 

12. Do you apply hand washing before and after cleaning the genital area?

Yes

No

13. The preferred underwear to wear is

Cotton and polyester 

Cotton 

Polyester 

14. The preferred underwear to wear is

Tight

Wide

Comfortable

15. Changing underwear interval is

Everyday

Every 2 days

Every week

More than one per day if needed

16. The time interval of genitalia hair removal

Every 20 days a

Every month

Every 2 months

More than 2 months
